# Hypertension and Postoperative Outcomes: A Retrospective Cohort Study

**DOI:** 10.1155/anrp/1180205

**Published:** 2026-07-15

**Authors:** Taeko Fukuda, Norihiko Inoue, Kazushi Maruo, Hiromasa Horiguchi

**Affiliations:** ^1^ Department of Anesthesiology, Institute of Medicine, University of Tsukuba, 1-1-1 Tenno-dai, Tsukuba City, 305-8575, Ibaraki, Japan, tsukuba.ac.jp; ^2^ Kasumigaura Medical Center Hospital (Tsuchiura Clinical Education and Training Center), National Hospital Organization, 2-7-14 Shimotakatu, Tsuchiura City, 300-8585, Ibaraki, Japan, hosp.go.jp; ^3^ Department of Clinical Data Management and Research, Clinical Research Center, National Hospital Organization Headquarters, 2-5-21 Higashigaoka, Meguro-ku, 152-8621, Tokyo, Japan, tokyo.ac.jp; ^4^ Department of Health Policy and Informatics, Graduate School of Medical and Dental Sciences, Institute of Science Tokyo, 1-5-45 Yushima, Bunkyo-ku, 113-8519 Tokyo, Japan, tokyo.ac.jp; ^5^ Institute of Clinical Epidemiology (iCE), Showa University, 1-5-8 Hatanodai, Shinagawa-ku, 142-8555, Tokyo, Japan, tokyo.ac.jp; ^6^ Department of Biostatistics, Faculty of Medicine, University of Tsukuba, 1-1-1 Tenno-dai, Tsukuba City, 305-8575, Ibaraki, Japan, tsukuba.ac.jp

## Abstract

**Background:**

The prevalence of hypertension is high. However, hypertensive patients often remain undiagnosed or poorly controlled, posing challenges for anesthesiologists before surgery. Although well‐established guidelines have been proposed, some uncertainties remain. This study aimed to test the hypothesis that the closer the preoperative blood pressure (BP) is to normal, the lower the incidence of unfavorable postoperative outcomes in hypertensive patients using data from 2016 onward.

**Methods:**

Medical records of adult patients under general anesthesia for surgery at 68 hospitals in Japan between 2016 and 2021 were reviewed. In accordance with World Health Organization criteria, patients were categorized based on their maximum BP recorded the day before surgery into five groups: true normotension, controlled hypertension (patients with prior diagnosis and/or treatment of hypertension), and Stages 1, 2, and 3 hypertension groups. Postoperative mortality rates and incidence of unfavorable outcomes, including ischemic heart disease, cerebrovascular disease, aortic dissection, and acute renal failure, were compared using multivariable logistic regression analysis.

**Results:**

Data from 272,594 patients (either sex, aged between 20 and 106 years) were analyzed, excluding those undergoing cardiac, brain, and obstetric surgery. Mortality did not differ significantly between the four hypertension groups and the true normotension group. Compared with the true normotension group, the odds of ischemic heart disease were higher in the controlled, Stage 2, and Stage 3 hypertension groups. The odds of cerebrovascular disease and acute renal failure increased with hypertension stage, whereas the odds of aortic dissection were elevated only in Stage 3 hypertension.

**Conclusions:**

Our results showed that the closer preoperative BP was to normal, the better the postoperative outcomes for cerebrovascular disease and acute renal failure, but not for mortality, ischemic heart disease, or aortic dissection. Since the risk in the controlled hypertension group was higher than initially expected, our hypothesis was rejected.

## 1. Introduction

An estimated 1.28 billion people worldwide suffer from hypertension, with 46% unaware of their condition and only 42% receiving a diagnosis and treatment [1]. Furthermore, even when diagnosed with hypertension, many patients are unable to achieve adequate blood pressure (BP) control [2]. Therefore, anesthesiologists often encounter patients with uncontrolled hypertension just before surgery.

Recent guidelines advise against postponing surgery for patients with Stage 1 (systolic BP [SBP]: 140–159 and/or diastolic BP [DBP]: 90–99 mmHg) and Stage 2 (SBP: 160–179 and/or DBP: 100–109 mmHg) hypertension while advocating for emergency surgery even for Stage 3 cases (SBP: ≥ 180 and/or DBP: ≥ 110 mmHg) [3, 4]. However, the level of recommendation in the guidelines ranges from IIb (weak) to III (no recommendation) because they are based on small‐scale studies and a meta‐analysis of 30 observational studies published between 1971 and 2001 [5]. Recent advancements in monitoring and antihypertensive drugs may enable adequate management of abnormal BP and drastic fluctuations in BP during surgery. Surgical risks associated with the stage of hypertension may differ from those previously observed.

Several reviews have also reported a lack of clear evidence regarding the relationship between preoperative hypertension and postoperative outcomes [6–8]. To the best of our knowledge, six large‐scale studies investigating hypertension and postoperative outcomes have been conducted since 2000 (Table 1) [9–14]. However, half these studies used a less stringent definition of “hypertension requiring medication.” The remaining three studies were limited in scope, being confined to patients with hypertension, assessing solely mortality or ischemic heart diseases, or restricting findings to patients with preexisting cardiac risk. Although preoperative evaluation should fundamentally be comprehensive, clinical information obtained from simple scores such as hypertension staging may also be useful.

**TABLE 1:** Summary of previously published studies.

Author		Patient	*N*	Surgery	Definition of hypertension	Evaluation target	Reference
Present study		All patients	272,594	Elective noncardiac, non‐neurologic, non‐obstetric surgery	Stage 1 (SBP:140‐159, DBP:90–99) Stage 2 (SBP:160‐179, DBP:100–109) Stage 3 (SBP:180+, DBP:110+)^∗^	Mortality (in‐hospital), cardiovascular, renal, and neurologic morbidity	

Abdelmalak et al.	2018	All patients (cardiac risk)	58,276 (10,512)	Elective noncardiac surgery	Stage 1 (SBP:140‐159, DBP:90–99) Stage 2 (SBP:160‐179, DBP:100–109) Stage 3 (SBP:180+, DBP:110+)^∗^	Mortality (in‐hospital), cardiovascular, renal, and neurologic morbidity	[9]
Venkatesan et al.	2017	All patients	251,567	Elective noncardiac surgery	Numerical value	Mortality (30‐day)	[10]
Mathis et al.	2013	Outpatients	244,397	Day surgery	Hypertension requiring medication	Mortality and morbidity (3‐day)	[11]
Mashour et al.	2011	All patients	523,059	Noncardiac, non‐neurologic surgery	Hypertension requiring medication	Mortality (30‐day), stroke	[12]
Wax et al.	2010	All patients (hypertension)	209,985 (21,126)	Elective surgery	Preinduction BP:> 140/90 mmHg	Mortality (in‐hospital), elevated troponin, cancellation of surgery	[13]
Kheterpal et al.	2009	All patients	75,952	General surgery	Hypertension requiring medication	Mortality, acute kidney injury (30‐day)	[14]

Abbreviations: BP = blood pressure, DBP = diastolic blood pressure, SBP = systolic blood pressure.

^∗^This is based on the definition of hypertension in the European Society of Cardiology (ESC) and European Society of Hypertension (ESH) guidelines [9].

The rationale behind postponing surgery in patients with uncontrolled hypertension lies in the assessment of organ damage and control of BP [15–18]. While the importance of preoperative assessment of organ damage in hypertensive patients is well established (recommended level I) [3], a recent expert opinion study reported insufficient evidence to support that controlling BP in the preoperative period reduces risk [7]. Weksler et al. [19] reported no significant differences in postoperative outcomes between groups with and without BP control immediately before surgery in 989 patients with hypertension. Therefore, investigating a larger patient population to assess whether hypertensive patients with normalized BP before surgery are less likely to experience unfavorable postoperative outcomes compared to those with uncontrolled BP would be highly meaningful.

We hypothesize that the closer the preoperative BP is to normal, the lower the incidence of unfavorable postoperative outcomes in hypertensive patients. The purpose of this study was to test the above hypothesis using data from 2016 onwards and provide valuable insights into anesthetic management in hypertensive patients.

## 2. Methods

### 2.1. Ethical Approval

Following the Japanese ethical guidelines for human medical research, which are based on the Declaration of Helsinki, this study was approved by the Ethical Review Board of the University of Tsukuba (#1784) and registered under UMIN000048109 (June 20, 2022). To protect patient privacy, all personal identification data were encrypted in a secure room at the National Hospital Organization Headquarters. The requirement for informed consent was optional for this study, as determined by the institutional review board, due to the use of deidentified data.

### 2.2. Data Sources

This retrospective cohort study was conducted using data from 68 hospitals, accessed through the Diagnosis Procedure Combination (DPC) administrative claims database, spanning from January 1, 2016, to December 31, 2021. The DPC database is a diagnosis‐dominant, case‐mix system managed by the Ministry of Health, Labor, and Welfare, which links to a lump‐sum payment system [20]. Data extracted from the DPC database comprised demographic information such as age, sex, height, weight, smoking status, primary diagnosis, surgical procedure, functional status (based on activities of daily living [ADLs] at admission), hospital size, and comorbidities. Vital sign information was extracted by linking the DPC database with hospital electronic medical records using patient IDs, which were subsequently removed before analysis to protect patient privacy.

### 2.3. Selection of Patients and Variables

Adult patients (either sex, aged ≥ 20 years) who underwent surgery under general anesthesia during the study period were included, whereas those undergoing neurosurgery, cardiac or large vascular surgery, obstetric surgery, and same‐day surgery were excluded in line with previously published papers [9–14]. In the medical insurance system in Japan, the hospitalization management fee paid to hospitals is automatically reduced if the length of hospital stay exceeds 14 days. Therefore, in cases of hospitalization for surgery, the preoperative length of hospital stay is usually within 14 days. Cases in which the preoperative hospitalization period exceeded 14 days were excluded from this study, as they were considered unexpected emergency surgery (e.g., bleeding, fracture due to falls). Since our medical insurance system does not specify a clear rule for the preoperative length of hospital stay for elective surgery, we adopted the above criteria instead. Patients with insufficient data or hypotension (SBP < 90 mmHg and/or DBP < 60 mmHg) were also excluded. Eligible patients were categorized into five groups based on the maximum SBP and/or DBP recorded on the day before surgery, according to established guidelines, including true normotension (SBP: 90–139 and/or DBP: 60–89 mmHg), controlled hypertension, as well as Stage 1 (SBP: 140–159 and/or DBP: 90–99 mmHg), Stage 2 (SBP: 160–179 and/or DBP: 100–109 mmHg), and Stage 3 (SBP: ≥ 180 and/or DBP: ≥ 110 mmHg) hypertension groups. The controlled hypertension group was defined as patients with preoperative normotension who were on antihypertensive medications and/or had a diagnosis of hypertension at the time of admission [16, 21, 22]. To investigate the dose‐dependent relationship between postoperative outcomes and the degree of preoperative hypertension, we used the World Health Organization (WHO) and European hypertension categories, which divide SBP and DBP into 20 and 10 mmHg increments, respectively. Some patients in the Stage 1 hypertension group were initially speculated to have white‐coat hypertension. However, recent studies have highlighted that even with white‐coat hypertension, arteriosclerosis progresses, leading to an increased risk of cardiovascular disease [23–25]. Therefore, we decided to adopt this definition for our study.

Although body mass index (BMI) and heart rate (HR) are continuous variables, they were converted to categorical variables, as both high and low values can be predictive of risk. BMI was classified into four categories according to WHO standards [26]. Tachycardia and bradycardia were defined as HR of more than 100 and less than 60 beats/minute, respectively [27]. Smoking habits were classified into smoking and nonsmoking, and those who had quit smoking were included as smokers. Surgical procedures were classified into five categories: general surgery, orthopedics, gynecology, urology, and other specialties. Regarding the type of surgery, four departments with the highest numbers of surgical cases were selected, and the remaining departments were grouped together as “other”. The “other” category included ophthalmology, otolaryngology, dermatology, and dentistry, which had relatively fewer cases performed under general anesthesia. Since differences in postoperative care proficiency were expected between departments managing a large number of hypertensive patients and those managing fewer, the five surgical categories were included as independent variables. The functional status in the DPC database included the following 10 ADLs: eating, transferring, grooming, toileting, bathing, walking on a flat surface, using stairs, dressing, defecating, and micturating [28]. Three of the 10 items (eating, walking on a flat surface, and defecating), which were assumed to be particularly closely related to nutritional status, daily activity level, and bedridden status, were used as independent factors [29, 30]. Hospitals with fewer than 400 beds were classified as small hospitals. According to Japanese standards, a hospital that provides advanced medical care must have 400 or more beds. Comorbidities were identified using the International Statistical Classification of Diseases and Related Health Problems, 10th revision, code (ICD‐10). After two items related to malignant tumors were combined into a single item and acquired immunodeficiency syndrome was excluded, the remaining 15 items from the 17‐item Charlson Comorbidity Index [31] were used to classify comorbidities. Since the severity of chronic pulmonary and renal diseases was unclear, the presence or absence of preoperative oxygen therapy and hemodialysis was included as an independent variable. Approximately 27% of patients had a preoperative hospital stay of more than 3 days. Although the reason for this long preoperative hospitalization was unknown, some risk may have been involved. Therefore, the number of hospitalization days before surgery was also included as an independent variable. Anesthesia time, defined in the Japanese medical insurance system as the period during which anesthesiologists manage patients using the anesthesia machine, was used as an alternative variable for surgical duration. In addition to surgical time, anesthesia time includes the time required to prepare invasive monitors or lines, and therefore partly reflects the complexity of surgery. The maximum BP and HR were defined as the highest values recorded the day before surgery. BP and HR were measured by nurses using automatic sphygmomanometers.

### 2.4. Outcomes

Study outcomes included in‐hospital mortality and the presence or absence of postoperative complications, including ischemic heart disease (I20–I24), cerebrovascular disease (I60–I69), aortic dissection (I71, I72), and acute renal failure (N17), as indicated by the ICD‐10 codes in the database. Based on the recorded dates of diagnosis, we selected only complications diagnosed postoperatively, not those identified during hospitalization. For angina (I20), comorbidities were checked to exclude chronic angina. Patients who started hemodialysis the day after surgery were counted as cases of acute renal failure, even if no diagnosis had been made. In‐hospital mortality was defined as death in the first hospital; therefore, deaths after transfer to another hospital were not included.

### 2.5. Statistical Analysis

Multivariable logistic regression models incorporating the following 30 independent variables: age, sex, BMI, smoking habits, functional status (eating, walking on a flat, and defecating), clinical department, hospital scale, 15 comorbidities, preoperative oxygen therapy and hemodialysis, number of preoperative days, and anesthesia time, as well as HR and BP the day before surgery, were utilized to analyze mortality rates and morbidities. The goodness of fit of these models was assessed using C‐statistics. Odds ratios (ORs) with 95% confidence intervals [95% CIs] were calculated for each hypertension group. The level of statistical significance for all tests was set to 0.05. Statistical analyses were performed using SPSS, Version 26 (IBM, Armonk, NY, USA).

## 3. Results

### 3.1. Selection Criteria and Characteristics of Participants

**FIGURE 1:**
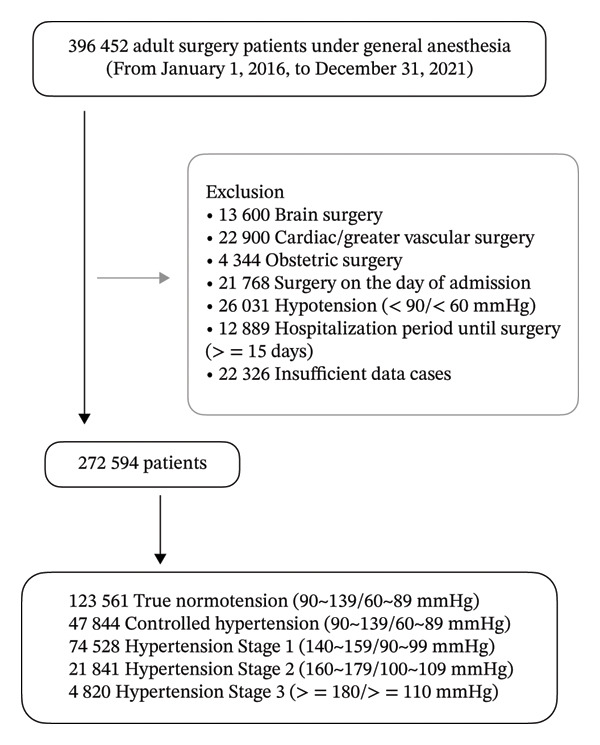
Flow diagram of inclusion and exclusion of patients for the analysis of preoperative blood pressure and postoperative outcomes.

A total of 396,452 patients aged ≥ 20 years who underwent surgery under general anesthesia were initially identified. After patients with missing data (*n* = 22,326) were excluded and exclusion criteria were applied, the final analysis was conducted on 272,594 cases (Figure 1). The proportions of patients with true normotension, controlled hypertension, as well as Stage 1, Stage 2, and Stage 3 hypertension, were approximately 45%, 18%, 27%, 8%, and 2%, respectively. The number of patients in the departments of general surgery, orthopedics, gynecology, urology, and other specialties was 115,491 (42%), 72,629 (27%), 31,636 (12%), 27,077 (10%), and 25,761 (10%), respectively. The proportion of hypertensive patients in each clinical department was approximately 63% in orthopedics and urology, 55% in general surgery, 48% in other specialties, and 35% in gynecology. Table 2 shows the demographic characteristics of the patients. The median preoperative hospital stay was 2 days in the controlled hypertension group and 1 day in the other groups.

**TABLE 2:** Demographic data (*n* = 272,594).

Variables		NT	Cont_HT	HT_St 1	HT_St 2	HT_St 3
*N*		123,561	47,844	74,528	21,841	4820
Age		58 ± 17	72 ± 11	67 ± 14	69 ± 14	67 ± 15
	< 60	59,951 (48.5%)	5530 (11.6%)	19,004 (25.5%)	5166 (23.7%)	1423 (29.5%)
	60–79	52,503 (42.5%)	30,479 (63.7%)	42,014 (56.4%)	11,926 (54.6%)	2321 (48.2%)
	≥ 80	11,107 (9.0%)	11,835 (24.7%)	13,510 (18.1%)	4749 (21.7%)	1076 (22.3%)

Sex (M/F)	Male	51,292 (41.5%)	25,149 (52.6%)	36,338 (48.8%)	10,665 (48.8%)	2459 (51.0%)
BMI (kg/m^2^)						
Under	< 18.5	13,210 (10.7%)	3285 (6.9%)	5100 (6.8%)	1589 (7.3%)	423 (8.8%)
Normal	18.5–25	80,549 (65.2%)	27,131 (56.7%)	42,978 (57.7%)	12,228 (56.0%)	2542 (52.7%)
Over	≥ 25	24,207 (19.6%)	13,754 (28.7%)	20,691 (27.8%)	6174 (28.3%)	1352 (28.0%)
Obesity	≥ 30	5595 (4.5%)	3674 (7.7%)	5759 (7.7%)	1850 (8.5%)	503 (10.4%)
Smoking	+	41,171 (33.3%)	17,007 (35.5%)	25,209 (33.8%)	7131 (32.6%)	1573 (32.6%)
Clinical departments						
General		52,339 (42.4%)	22,213 (46.4%)	30,746 (41.3%)	8443 (38.7%)	1750 (36.3%)
Ortho		26,918 (21.8%)	14,604 (30.5%)	22,115 (29.7%)	7283 (33.3%)	1709 (35.5%)
Gyne		20,692 (16.7%)	2185 (4.6%)	6792 (9.1%)	1591 (7.3%)	376 (7.8%)
Uro		10,116 (8.2%)	5479 (11.5%)	8341 (11.2%)	2641 (12.1%)	500 (10.4%)
Other		13,496 (10.9%)	3363 (7.0%)	6534 (8.8%)	1883 (8.6%)	485 (10.1%)
Hospital scale	< 400 beds	23,731 (19.2%)	9627 (20.1%)	15,451 (20.7%)	4366 (20.0%)	865 (17.9%)
ADL dependent						
Eating	+	2543 (2.1%)	1802 (3.8%)	2549 (3.4%)	1215 (5.6%)	427 (8.9%)
Walking	+	5246 (4.2%)	4140 (8.7%)	5360 (7.2%)	2334 (10.7%)	748 (15.5%)
Defecating	+	3824 (3.1%)	2971 (6.2%)	3984 (5.3%)	1826 (8.4%)	583 (12.1%)
Heart rate	< 60	6511 (5.3%)	3286 (6.9%)	4016 (5.4%)	1083 (5.0%)	181 (3.8%)
	> 100	4290 (3.5%)	1688 (3.5%)	3656 (4.9%)	1504 (6.9%)	611 (12.7%)

CCI1_MI	+	462 (0.4%)	945 (2.0%)	596 (0.8%)	195 (0.9%)	46 (1.0%)
CCI2_CVD	+	2001 (1.6%)	3114 (6.5%)	2882 (3.9%)	939 (4.3%)	254 (5.3%)
CCI3_CHF	+	1948 (1.6%)	3450 (7.2%)	2576 (3.5%)	853 (3.9%)	228 (4.7%)
CCI4_Rheum	+	1762 (1.4%)	1076 (2.2%)	1456 (2.0%)	446 (2.0%)	89 (1.8%)
CCI5_Dementia	+	1485 (1.2%)	1621 (3.4%)	1816 (2.4%)	823 (3.8%)	219 (4.5%)
CCI6 _DM	+	11,078 (9.0%)	11,066 (23.1%)	10,847 (14.6%)	3178 (14.6%)	656 (13.6%)
CCI7 Liver D (mild)	+	3633 (2.9%)	2609 (5.5%)	2907 (3.9%)	887 (4.1%)	214 (4.4%)
CCI8_Ulcer	+	8675 (7.0%)	4760 (9.9%)	5409 (7.3%)	1529 (7.0%)	313 (6.5%)
CCI9_PVD	+	673 (0.5%)	1085 (2.3%)	853 (1.1%)	320 (1.5%)	84 (1.7%)
CCI10_CPD	+	4615 (3.7%)	3273 (6.8%)	3566 (4.8%)	858 (3.9%)	181 (3.8%)
CCI11 & 15_Cancer	+	54,681 (44.3%)	24,065 (50.3%)	32,749 (43.9%)	9027 (41.3%)	1809 (37.5%)
CCI12_DM (comp)	+	1103 (0.9%)	1466 (3.1%)	1449 (1.9%)	581 (2.7%)	182 (3.8%)
CCI13_Plegia	+	169 (0.1%)	126 (0.3%)	133 (0.2%)	52 (0.2%)	20 (0.4%)
CCI14_Renal D	+	849 (0.7%)	1472 (3.1%)	1681 (2.3%)	1040 (4.8%)	413 (8.6%)
CCI16_Liver D(ms)	+	144 (0.1%)	150 (0.3%)	109 (0.1%)	29 (0.1%)	7 (0.1%)
O_2__before	+	13,911 (11.3%)	9520 (19.9%)	10,708 (14.4%)	3294 (15.1%)	756 (15.7%)
HD_before	+	255 (0.2%)	349 (0.7%)	617 (0.8%)	579 (2.7%)	306 (6.3%)
Pre‐Ope days		1 (1, 2)	2 (1, 4)	1 (1, 3)	1 (1, 2)	1 (1, 2)
Anesthesia time (minutes)		190 ± 115	214 ± 126	196 ± 115	190 ± 113	187 ± 110

*Note:* HT_St 1: Hypertension Stage 1 group, HT_St 2: Hypertension Stage 2 group, HT_St 3: Hypertension Stage 3 group, M: male, F: female, Ortho: orthopedics, Gyne: gynecology, Uro: urology, DM: diabetes, Liver D (mild): liver disease (mild), Ulcer: peptic ulcer disease, Rhuem: rheumatic disease, DM (comp): diabetes with complications, Plegia: paraplegia or hemiplegia, Renal D: renal disease. Liver D (ms): liver disease (moderate or severe), O_2__before: oxygen therapy before surgery, HD_before: hemodialysis before surgery, Pre‐Ope days: median number of days of preoperative hospitalization (25th, 75th percentiles).

Abbreviations: ADL = activities of daily living, BMI = body mass index, CCI = Charlson Comorbidity Index, CHF = congestive heart failure, Cont_HT = controlled hypertension group, CPD = chronic pulmonary disease, CVD = cerebrovascular disease, MI = myocardial infarction, NT = true normotension group, PVD = peripheral vascular disease.

### 3.2. In‐Hospital Mortality and Postoperative Complications of Each Group

**FIGURE 2:**
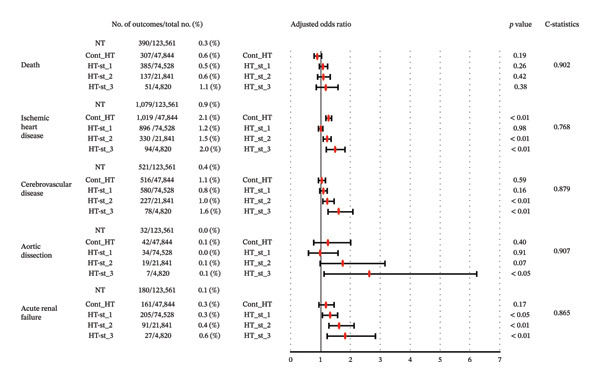
Adjusted odds ratio for mortality and other outcomes across hypertension groups. The adjusted odds ratios were derived from multivariable logistic regression models, with independent variables including age, sex, body mass index, smoking habits, functional status (eating, walking, and defecating), clinical department, hospital scale, 15 comorbidities, preoperative oxygen therapy and hemodialysis, number of preoperative days, and anesthesia time, as well as maximum heart rate and blood pressure on the day before surgery. The true normotension group served as the reference for calculating the odds ratios. NT: true normotension group; Cont_HT: controlled hypertension group; HT‐st_1: Stage 1 hypertension group; HT‐st_2: Stage 2 hypertension group; HT‐st_3: Stage 3 hypertension group; CI: confidence interval.

Multivariable logistic regression analysis revealed no significant difference in the OR for mortality between the four hypertension groups and the true normotension groups. However, compared with the true normotension group, the incidence of ischemic heart disease was significantly higher in the controlled hypertension, as well as Stage 2, and Stage 3 hypertension groups (OR [95% CI]: 1.27 [1.15–1.39]; *p* < 0.01, 1.22 [1.07–1.38]; *p* < 0.01, and 1.48 [1.18–1.85]; *p* < 0.01, respectively). The incidence of cerebrovascular disease was significantly elevated in the Stage 2 and Stage 3 hypertension groups (OR [95% CI]: 1.25 [1.06–1.49]; *p* < 0.01, and 1.62 [1.24–2.10]; *p* < 0.01, respectively) compared with that in the true normotension group. A higher incidence of aortic dissection was observed in the Stage 3 hypertension group (OR [95% CI]: 2.63 [1.11–6.25]; *p* < 0.05). The incidence of acute renal failure was higher in the Stage 1, Stage 2, and Stage 3 hypertension groups (OR [95% CI]: 1.30 [1.05–1.60]; *p* < 0.05, 1.64 [1.26–2.15]; *p* < 0.01, and 1.86 [1.20–2.86]; *p* < 0.01, respectively) compared with that in the true normotension group, with severity appearing to be proportional to the hypertension stage. Figure 2 shows the unadjusted results and provides a graphical representation of the adjusted results.

## 4. Discussion

As predicted, most outcomes in patients with hypertension were worse than those in patients with true normotension. Notably, patients with Stage 3 hypertension had significantly higher odds of all unfavorable outcomes except mortality. However, patients with hypertension whose BP normalized before surgery also had higher odds of ischemic heart disease. This unexpected result contradicted our hypothesis that achieving BP closer to normal would lead to improved postoperative outcomes.

We observed no significant disparity in mortality between the four hypertension groups and the true normotension group. One plausible explanation is that factors beyond the circulatory system, such as cancer progression or infection, may contribute to mortality. The incidence of ischemic heart disease was significantly higher in the Stage 2 and Stage 3 hypertension groups compared with that in the true normotension group, with an unexpectedly high OR in the controlled hypertension group. Several studies have associated a lower DBP with cardiovascular events [32, 33]. Slightly elevated BP, particularly DBP, maintains coronary blood flow and may confer advantages against ischemic heart disease. The ORs for cerebrovascular disease were significantly higher in the Stage 2 or higher hypertensive groups compared with those in the true normotensive group. For patients deemed to have a high cerebrovascular risk based on medical history or family background, it is necessary to assess the risk of hypertension comprehensively, without relying solely on preoperative BP. Regarding aortic dissection, a higher OR was observed in the Stage 3 hypertension group. There are two possible explanations for the higher odds of aortic dissection observed in the controlled hypertension group, despite the lack of statistical significance. The controlled hypertension group may have included patients requiring strict BP control due to preexisting aortic conditions, or those with severe hypertension whose BP had reached normotensive levels due to being bedridden following severe frailty [34]. The hypertension groups exhibited significantly higher ORs for acute renal failure than the true normotension group, with the ORs appearing to correlate with severity. Kidney damage, a common complication of essential hypertension, typically develops 5–10 years after the onset of hypertension; however, it is frequently unnoticed due to its asymptomatic nature [1, 35]. The increase in OR in proportion to the severity of hypertension strongly suggests that organ damage may be present even with mild hypertension.

The risk in the controlled hypertension group was higher than initially expected. BP reportedly decreases when physical activity is reduced due to hospitalization or similar factors [36, 37]. In addition to patients with well‐controlled hypertension, this group may have included patients whose BP decreased as a result of hospitalization or reduced activity due to physical illness. As shown in Table 2, the controlled hypertension group had a median preoperative hospital stay of 2 days, with the widest distribution among all groups. The number of preoperative hospitalization days was used as an independent variable to adjust for the effects of hospitalization. However, identifying a factor that directly reflected the level of physical activity was difficult. The methods available to identify patients with severely restricted ADLs due to conditions such as physical weakness, lower extremity edema, or respiratory problems were limited. In this study, we used three ADL items (eating, walking, and defecating), which are considered closely related to physical activity level, as independent variables. However, some patients remained in bed, even though they were able to walk independently. Dependence in ADLs and reduction in physical activity are not always proportional. Hidden hypertension carries a high risk; therefore, understanding the underlying factors contributing to normalization of BP is important.

BMI and HR were analyzed as continuous variables. However, as the main results were the same whether they were analyzed as continuous or categorical variables, BMI and HR were presented as categorical variables for easier clinical interpretation. Since preoperative hemodialysis and oxygen therapy were suspected to be strongly associated with the outcomes, a logistic regression analysis was performed excluding these two variables. The c‐statistics for mortality, ischemic heart disease, and aortic dissection were 0.898, 0.718, and 0.901, respectively, which were only slight differences compared to the original values of 0.901, 0.758, and 0.902. Furthermore, the c‐statistics for cerebrovascular disease and acute renal failure remained unchanged. Therefore, preoperative dialysis and oxygen therapy are important predictors, but are not strong enough to nullify the significance of the regression analysis. The complexity and risk of surgical procedures affect postoperative outcomes. However, accurately identifying and parameterizing “major surgery” among more than 1000 surgical procedure codes was difficult because the definition of major surgery is multifactorial, including vascular clamping or organ ischemia, intraoperative blood loss, high noradrenaline requirements, prolonged operative time, and perioperative blood transfusion [38]. Because anesthesia records were not available, the amount of blood loss, transfusion, catecholamine administered, and operative duration were unknown. Anesthesia time was used as a proxy variable for the duration of surgery. Although anesthesia time reflects surgical complexity to some extent, it only partially represents surgical risk, limiting the interpretation of the results.

Despite our promising results, our study has several limitations, including its retrospective study design. First, data were gathered in an environment where high BP guidelines were widely known, resulting in a small number of Stage 3 hypertension cases. Second, only patients who underwent surgery were included, excluding those whose procedures were canceled, leaving it unclear whether surgery was postponed for the management of BP. Third, the DPC database does not include information indicating emergency surgery. Because of numerous exceptions, such as transportation by family members or daytime emergency surgery on weekdays, it was difficult to identify emergency surgeries using information on ambulance use or the date and time the surgery was performed. Therefore, all patients who underwent surgery on the day of admission were excluded. Consequently, some patients who underwent nonemergency surgery may also have been excluded from the study. Fourth, data on antihypertensive medications were incomplete in this study. Fifth, due to the nature of the data used, adjustment for the risk of surgical procedures was not possible, and the severity of both comorbidities and outcomes could not be assessed. Sixth, since anesthesia records are not included in the DPC database, intraoperative events such as hypotension and/or hypertension, blood loss, and dehydration and/or anemia, and data on immediate preoperative BP were unavailable. Since preoperative BP often fluctuates due to stress and other factors, using data from the day before surgery was considered appropriate. Seventh, because the study population was predominantly from a single country, the generalizability of these findings to other regions of the world remains uncertain. In particular, the length of preoperative hospital stay varies across national insurance systems, which may affect the interpretation of the results. Further research is warranted to address these limitations and confirm our findings.

## 5. Conclusion

Hypertension is highly prevalent and can significantly impact anesthesia management, necessitating comprehensive preoperative evaluation. This study showed that a higher stage of hypertension was associated with increased risks of cerebrovascular disease and acute renal failure. However, no clear trend was observed in which the odds of mortality, ischemic heart disease, or aortic dissection increased in proportion to the stage of hypertension. The controlled hypertension group was speculated to include patients whose BP had normalized due to reduced daily activity caused by hospitalization or physical weakness, or due to strict antihypertensive treatment for a dangerous condition. This study rejected the initial simplistic hypothesis and suggested that the true normotension group and the controlled hypertension group cannot be considered identical. Further large‐scale observational studies with more detailed data or prospective studies are needed to confirm these findings.

## Author Contributions

Taeko Fukuda: conceptualization, data curation, formal analysis, funding acquisition, investigation, methodology, project administration, visualization, writing–original draft, and writing–review and editing.

Norihiko Inoue: conceptualization, funding acquisition, and supervision.

Kazushi Maruo: formal analysis and methodology.

Hiromasa Horiguchi: data curation and resources.

## Funding

This study was supported by the Japan Society for the Promotion of Science (grant number: 23K09571).

## Conflicts of Interest

The authors declare no conflicts of interest.

## Data Availability

Data are available from the Department of Clinical Data Management and Research, Clinical Research Center, National Hospital Organization Headquarters, upon reasonable request from the last author.
